# The ICU environment contributes to the endemicity of the “*Serratia marcescens* complex” in the hospital setting

**DOI:** 10.1128/mbio.03054-23

**Published:** 2024-04-02

**Authors:** Sonia Aracil-Gisbert, Miguel D. Fernández-De-Bobadilla, Natalia Guerra-Pinto, Silvia Serrano-Calleja, Ana Elena Pérez-Cobas, Cruz Soriano, Raúl de Pablo, Val F. Lanza, Blanca Pérez-Viso, Sandra Reuters, Henrik Hasman, Rafael Cantón, Fernando Baquero, Teresa M. Coque

**Affiliations:** 1 Microbiology, Ramón y Cajal University Hospital and Ramón y Cajal Health Research Institute (IRYCIS), Madrid, Spain; 2 Member of the ESCMID Study Group for Epidemiological Markers (ESGEM), Basel, Switzerland; 3 Member of the ESCMID Food- and Water-borne Infections Study Group (EFWISG), Basel, Switzerland; 4 Biomedical Research Center Network of Infectious Diseases (CIBERINFEC), Madrid, Spain; 5 Intensive Medicine, Ramón y Cajal University Hospital and Ramón y Cajal Health Research Institute (IRYCIS), Madrid, Spain; 6 University of Alcalá (UAH), Madrid, Spain; 7 Bioinformatics Unit, Ramón y Cajal University Hospital and Ramón y Cajal Health Research Institute (IRYCIS), Madrid, Spain; 8 Institute for Infection Prevention and Control, Medical Center–University of Freiburg, Freiburg, Germany; 9 Statens Serum Institut, Copenhagen, Denmark; 10 Biomedical Research Center Network of Epidemiology and Public Health (CIBERESP), Madrid, Spain; McMaster University, Hamilton, Ontario, Canada

**Keywords:** *Serratia*, *Serratia marcescens*, *Serratia nevei*, AmpC, plasmid, IncL, VIM-1, hospital environment, “source-sink” dynamics

## Abstract

*Serratia marcescens* is an opportunistic pathogen historically associated with sudden outbreaks in intensive care units (ICUs) and the spread of carbapenem-resistant genes. However, the ecology of *S. marcescens* populations in the hospital ecosystem remains largely unknown. We combined epidemiological information of 1,432 *Serratia* spp. isolates collected from sinks of a large ICU that underwent demographic and operational changes (2019–2021) and 99 non-redundant outbreak/non-outbreak isolates from the same hospital (2003–2019) with 165 genomic data. These genomes were grouped into clades (1–4) and subclades (A and B) associated with distinct species: *Serratia nematodiphila* (1A), *S. marcescens* (1B), *Serratia bockelmannii* (2A), *Serratia ureilytica* (2B), *S. marcescens*/*Serratia nevei* (3), and *S. nevei* (4A and 4B). They may be classified into an *S. marcescens* complex (SMC) due to the similarity between/within subclades (average nucleotide identity >95%–98%), with clades 3 and 4 predominating in our study and publicly available databases. Chromosomal AmpC β-lactamase with unusual basal-like expression and prodigiosin-lacking species contrasted classical features of *Serratia*. We found persistent and coexisting clones in sinks of subclades 4A (ST92 and ST490) and 4B (ST424), clonally related to outbreak isolates carrying *bla*_VIM-1_ or *bla*_OXA-48_ on prevalent IncL/pB77-CPsm plasmids from our hospital since 2017. The distribution of SMC populations in ICU sinks and patients reflects how *Serratia* species acquire, maintain, and enable plasmid evolution in both “source” (permanent, sinks) and “sink” (transient, patients) hospital patches. The results contribute to understanding how water sinks serve as reservoirs of *Enterobacterales* clones and plasmids that enable the persistence of carbapenemase genes in healthcare settings, potentially leading to outbreaks and/or hospital-acquired infections.

**IMPORTANCE:**

The “hospital environment,” including sinks and surfaces, is increasingly recognized as a reservoir for bacterial species, clones, and plasmids of high epidemiological concern. Available studies on *Serratia* epidemiology have focused mainly on outbreaks of multidrug-resistant species, overlooking local longitudinal analyses necessary for understanding the dynamics of opportunistic pathogens and antibiotic-resistant genes within the hospital setting. This long-term genomic comparative analysis of *Serratia* isolated from the ICU environment with isolates causing nosocomial infections and/or outbreaks within the same hospital revealed the coexistence and persistence of *Serratia* populations in water reservoirs. Moreover, predominant sink strains may acquire highly conserved and widely distributed plasmids carrying carbapenemase genes, such as the prevalent IncL-pB77-CPsm (pOXA48), persisting in ICU sinks for years. The work highlights the relevance of ICU environmental reservoirs in the endemicity of certain opportunistic pathogens and resistance mechanisms mainly confined to hospitals.

## INTRODUCTION

The hospital environment, including surfaces and water systems, is increasingly recognized as a reservoir for multidrug-resistant *Enterobacterales*, constituting a risk factor for hospital-acquired infections (HAIs) and outbreaks (1). The Centers for Disease Control and Prevention’s Division of Healthcare Promotion encourages researchers to study the microbial behavior in hospital wards’ environmental reservoirs. The goal is to protect patients by gaining insights into how antibiotic-resistant genes, carried by “high-risk” clones and plasmids, emerge, persist, and evolve within and between “sources” (permanent, e.g., ICU environmental reservoirs) and “sinks” (transient, e.g., patients) hospital patches (2). The “source-sink” dynamic model is commonly used in ecology to describe how variation in “habitat” (defined as an array of resources and physical and biotic factors that are present in an area) quality can impact the growth or decline of microbial organisms (3) but has not been explored much at a clinical level (4, 5).

*Serratia*, a bacterial genus of the order *Enterobacterales* (6), accounts for about 1%–2% of HAIs and can cause outbreaks with high morbidity and mortality rates, especially in pediatric or intensive care unit (ICU) wards (7). According to the National Center for Biotechnology Information (NCBI) taxonomy database, the genus *Serratia* comprises 26 species heterogeneously distributed in water, soil, and the rhizosphere (8). These species may cause infections in humans, animals, or plants (7–9), with *Serratia marcescens* being the most common cause of human infections (7). Transmission of some human-associated *Serratia* species through contaminated medical devices such as nebulizers and catheters as well as via healthcare workers is well known (7), and they have also been isolated from water sinks (10–13). Recent phylogenetic studies have shown that some *Serratia* species, such as *S. marcescens, Serratia ureilytica*, or *Serratia nematodiphila*, are part of a larger group called “*Serratia marcescens* complex” (SMC), which highlights the remarkable diversification of *Serratia* populations (8, 14–16).

One of the main features of *S. marcescens* and other *Enterobacterales*, known as the ESCPM group (*Enterobacter* spp., *S. marcescens*, *Citrobacter freundii*, *Providencia* spp., *Morganella morganii*), is their intrinsic resistance to β-lactams [amino- and carboxy-penicillins, amoxicillin-clavulanate (AMC), first- and second-generation cephalosporins (1GC and 2GC)]. This resistance is attributed to the production of a chromosomally encoded inducible AmpC β-lactamase (17, 18). The induction of AmpC β-lactamase is of serious clinical concern as it increases the risk of AmpC derepression upon therapy (19). Such risk, combined with the increasing occurrence since the 1990s of plasmid-mediated extended-spectrum β-lactamases (ESBLs) that confer resistance to third-generation cephalosporins (3GCs), has led to the discouraged use of cephalosporins and the increased use of carbapenems for *Serratia* infections (20). Consequently, there has been a rise in carbapenem-resistant strains causing severe nosocomial infections (21).

In this work, we analyzed the diversity of *Serratia* in the environment of the largest ICU ward in our hospital, where repeated outbreaks of ESBL and carbapenemase *Enterobacterales* producers occurred over at least 20 years (22–25). Our study aimed to understand two main aspects: first, the role of the ICU environment in the endemicity of *Serratia* within the hospital [“source-sink” dynamics (4)], and second, the potential of *Serratia* as a host of clinically relevant plasmids that carry ESBL and/or carbapenemase genes.

## RESULTS

### Spatiotemporal distribution of *Serratia* spp. in the intensive care unit

The prospective long-term analysis of *Serratia* populations in the environmental reservoirs of the largest ICU ward of a tertiary hospital, which underwent demographic changes and was sampled during both post-outbreak and non-outbreak periods, offered a unique opportunity to analyze the impact of the ICU environment in the epidemiology of this nosocomial pathogen. Considering such demographic changes, the study was divided into three periods. Periods A (March 2019–February 2020) and B (July 2020–February 2021) covered the ICU occupancy before and during the SARS-CoV-2 epidemic, respectively. Period C (March 2021–December 2021) covered the sampling after the ICU patients were transferred to a renewed ward on another hospital floor and the previous ward was operationally transformed for other hospital uses.

**Fig 1:**
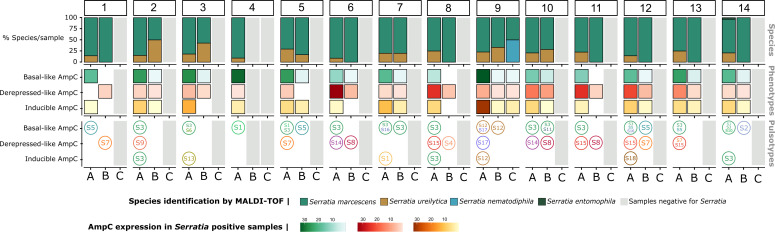
Spatiotemporal distribution of *Serratia* in sinks at Hospital Ramón y Cajal. Columns labeled by numbers embedded in boxes represent the 14 intensive care unit (ICU) rooms. Capital letters designate the study periods: A (March 2019–February 2020), B (July 2020–February 2021), and C (March 2021–December 2021), according to patient occupancy and use of the ICU hospital space, namely, ICU occupancy before (**A**) and after SARS-CoV lockdown (**B**), and patient clearing and conversion of the ward for other purposes unrelated to inpatient care (**C**). The first line shows the diversity and percentage of each *Serratia* species according to matrix-assisted laser desorption/ionization time of flight (MALDI-TOF) mass spectrometry. The second row reflects the diversity of AmpC phenotypes, each phenotype being represented by a color: (i) basal-like expression of the AmpC enzyme (AMC^S^ + FOX^S^ + 3 GC^S^); (ii) derepressed-like AmpC expression (AMC^R^ + FOX^R^ + 3GC^R^ +4 GC^R^ ± carbapenems^R^), and (iii) inducible AmpC β-lactamase expression (AMC^R^ + FOX^R^ + 3 GC^S^). The third row represents the most common XbaI digested genomic DNA patterns (pulsotypes, PT) of isolates with distinct AmpC phenotypes.

We identified 1,460 *Serratia* isolates from “wet” surfaces (448/1,126 samples, 39.8%; corresponding to sink drains, 253/448, 56.5%; washbasin surfaces, 168/448, 37.5%; and p-trap samples, 27/448, 6.0%) and from “dry” surfaces (19/1,291, 1.5%; 28 isolates). Figure 1 shows the spatiotemporal distribution of these isolates. The highest recovery rate of *Serratia* occurred during period A and overlapped with an ICU outbreak by *S. marcescens*-producing VIM-1 (385/746, 51.6%; 14/14 sinks). The isolation frequency drastically decreased during periods B and C (58/247, 23.5%, 13/14 sinks during period B; 5/133, 3.8%, 1/14 sinks during period C) (Fig. S1). Most of the colonies tested were preliminarily identified by matrix-assisted laser desorption/ionization-time of flight (MALDI-TOF) mass spectrometry (MS) as *S. marcescens* (*n* = 1,270, 88.7%), followed by *Serratia ureilytica* (*n* = 152, 10.6%), *Serratia nematodiphila* (*n* = 9, 0.6%), and *Serratia entomophila* (*n* = 1, 0.1%). *S. marcescens* and *S. ureilytica* isolates were found in all sinks, whereas *S. entomophila* was detected only once, and *S. nematodiphila* was isolated in a single room during period C. A similar sink colonization pattern by *Serratia* was observed in the sinks of the renewed ICU ward months after opening on a different floor (data not shown).

### *Serratia*’s β-lactam phenotype diversity

We identified three phenotypic patterns compatible with (i) basal-like expression of the AmpC enzyme [susceptibility (S) to AMC, FOX, 3GC, and fourth-generation cephalosporins (4GC): AMC^S^ + FOX^S^ + 3GC^S^ + 4 GC^S^]; (ii) inducible AmpC β-lactamase expression [resistance (R) to AMC, FOX, and 3GC^S^: AMC^R^ + FOX^R^ + 3GC^S^ + 4 GC^S^]; and (iii) derepressed-like AmpC expression (AMC^R^ + FOX^R^ + 3GC^R^ + 4GC^R^ ± carbapenems^R^) due to the presence of the carbapenemase VIM-1. We did not detect constitutively derepressed AmpC patterns (AMC^R^ + FOX^R^ + 3 GC^R^) in strains lacking *bla*VIM-1.

The first phenotype is similar to that of *Escherichia coli* but is exceptional for the ESCPM group and has not been reported for *S. marcescens* (18). Isolates with AmpC basal-like expression exhibited minimum inhibitory concentrations to aminopenicillins lower than those producing inducible AmpC (2–8 mg/mL vs 16/8 to >64 mg/mL, respectively).

All AmpC expression patterns were detected throughout the study, although isolates with the basal-like phenotype predominated (286/448 sink samples, 63.8%) over the “classical” AmpC phenotypes, either inducible (144/448, 32.1%) or derepressed-like (180/448, 40.2%). *Serratia* isolates exhibiting all three AmpC phenotypes were regularly detected in the same sink sample, although the frequency of each phenotype varied among sinks. A progressive decrease in the recovery of *Serratia* inducible phenotypes as well as an increase of derepressed-like AmpC isolates during period B, was observed (Fig. 1). All these phenotypes are still recovered from sinks in the space of the former ICU and the new ICU placed in another hospital floor (data not shown, ongoing studies). Resistance to chloramphenicol, gentamicin, and sulfamethoxazole-trimethoprim was observed only among AmpC derepressed-like *Serratia*, whereas susceptibility to ciprofloxacin and tetracycline varied significantly within and between groups. All 94 blood isolates showed an inducible AmpC phenotype, while the five outbreak isolates exhibited a derepressed-like AmpC phenotype attributed to the VIM-1 production.

### Genomic diversity of *Serratia* strains

#### The set of genomes analyzed

We selected a sample of 94/1,432 sink isolates for further typing/clustering analysis. The selection was randomly made among isolates representing the spatiotemporal distribution of species and phenotypes after eliminating redundancies (same antibiogram, morphology, and sampling site). Then, we preliminary analyzed the clonal relationship by pulsed-field gel electrophoresis (PFGE) typing and grouped the 94 isolates into 18 pulsotypes (PTs), of which 15 corresponded to *S. marcescens* and 3 to *S. ureilytica* strains according to MALDI-TOF. Some PTs, the predominant PT-S3 and PT-S1, PT-S5, PT-S7, PT-S14, and PT-S15, were detected in the majority of sinks during the whole study period, while others (PT-S2, PT-S4, PT-S6, PT-S9, PT-S10, PT-S12, PT-S13, PT-S17, and PT-S18) were persistently recovered in specific sinks (Fig. 1). Sixty-six isolates representing the spatiotemporal distribution, the diversity of β-lactam-phenotypes, and the PFGE patterns were fully sequenced.

To further explore the phylogenetic diversity and understand how variation in habitat quality influences the growth or decline of *Serratia* populations in the hospital environment [“the source-sink dynamics” ecological model (3)], we comparatively analyzed the genomes of 66 ICU-environmental isolates (64 from “wet” surfaces, two from “dry” surfaces) with 99 clinical isolates representing the epidemiology of *Serratia* in our institution in the last years. Most clinical isolates were collected in ICU wards, 94 caused independent episodes of bloodstream infection (BSI) (2003–2015), and five were recovered during outbreaks of VIM-1 and OXA-48 producers (2016–2018) (22, 25). The data set of genomic and epidemiological metadata of the 165 sequenced isolates is summarized in Table S1.

#### Phylogenomic analysis

**Fig 2:**
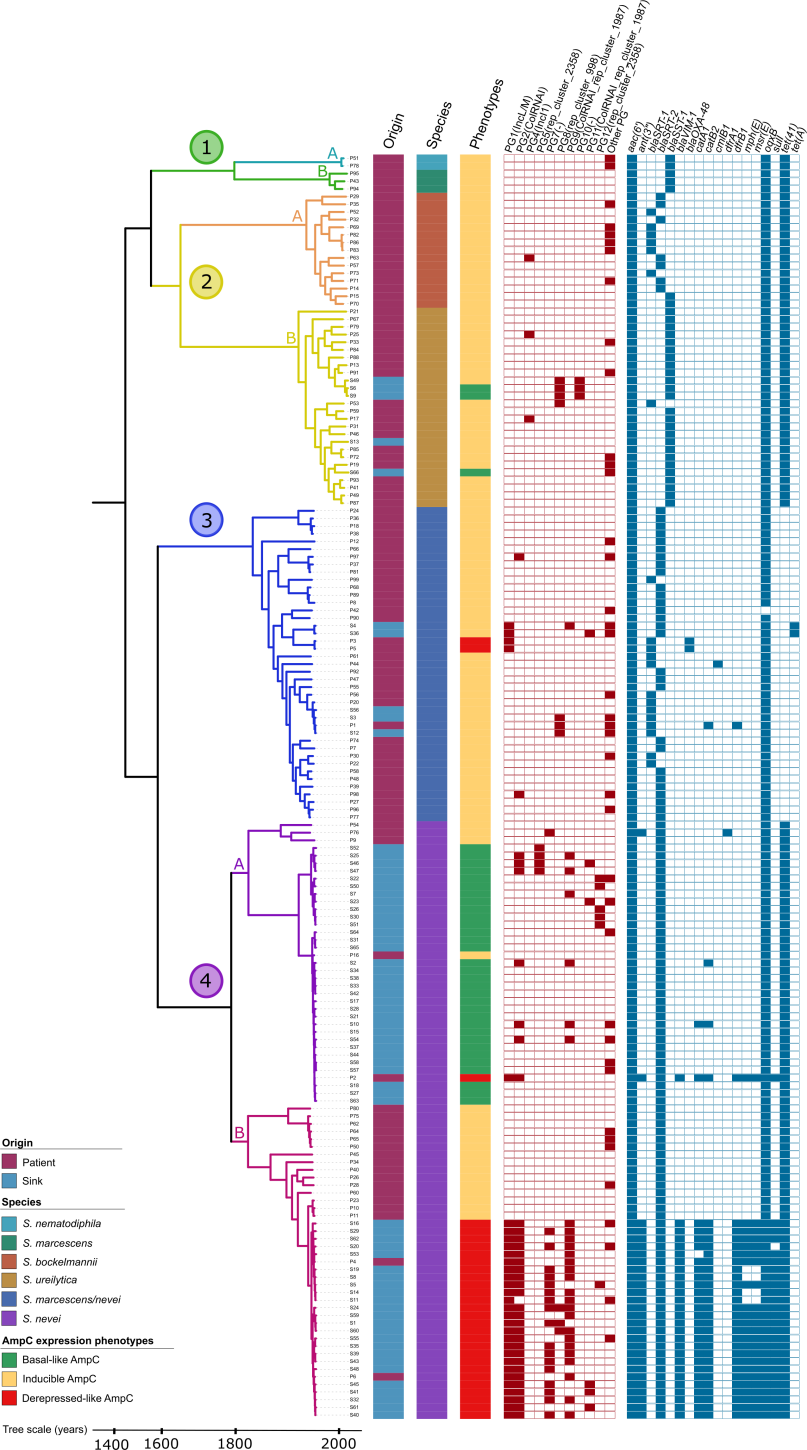
Phylogenetic structure of 165 isolates from the *Serratia marcescens* complex at Hospital Ramón y Cajal. A maximum likelihood tree was built with the 45,720 single nucleotide polymorphisms taken from the 2,583 core genes (appearing in 99% of the genomes). Strains were obtained from sinks and patients (stool, bloodstream, and outbreak related) from 2003 to 2020 (see Materials and Methods and Table S1). The main clades and subclades are shaded accordingly (see key). The origin of the sample (patient/sink), the species name according to genome similarity (*S. nematodiphila*, *S. marcescens*, *S. bockelmannii*, *S. ureilytica*, *S. marcescens/nevei*, and *S. nevei*), the AmpC phenotype expression (AmpC basal-like, AmpC inducible, AmpC extended-derepressed-like, no phenotype tested), the plasmid groups (PG, MOB-Typer), and the antibiotic resistance genes (ResFinder) are represented from left to right and labeled accordingly (see keys).

The chromosome length of the genomes analyzed ranged from 4.9 to 5.4 Mb (59.4% G + C content on average). We identified 2,583 core genes. The core genome-based phylogenetic tree represented in Fig. 2 shows four major clades (designated with Arabic numerals 1–4, with subclades defined by capital letters “A” or “B”). The correlation of each clade/subclade with known species was established by comparing these genomes with the Genome Taxonomic Database (GTDB) and the NCBI, which are considered the standardized microbial taxonomy based on genome phylogeny (26). Clade 1 split into subclades 1A and 1B, which corresponded with isolates identified as *S. nematodiphila* (Snm) and *S. marcescens* (Sm) [average nucleotide identity (ANI) >98%), and clade 2, into subclades 2A and 2B, corresponding to *Serratia bockelmannii* (Sb) and *S. ureilytica* (Su) (ANI >98%), respectively. Clade 3 was classified as *S. marcescens* or *Serratia nevei* (Sm/Snv) according to the GTDB (ANI >98%) or NCBI (ANI >95%) references, respectively. Clade 4 comprised genomes consistently identified as *S. nevei* (Snv) for GTDB or NCBI (ANI 96% and >97%, respectively) and split into subclades 4A and 4B. Discrepancies in the species assignment when using genome references of GTDB or NCBI were observed for subclade 1A (Snm vs Sm), subclade 2A (Sb vs Su), and clade 3 (Sm vs Snv), although the ANI values for any comparisons were very high (Table S1). For example, we observed values higher than 95% and increased to greater than 98% for genomes within each subclade (Fig. S2A). Ribosomal multilocus sequence typing (rMLST), a widely used diversity identifier, was also assessed as another method to establish the taxonomy (27), although it did not consistently reach the species level in most of our cases (Table S1). *Serratia* isolated from sinks were mostly grouped in clades 3 (Sm/Snv) and 4 (Snv), with a few isolates interspersed in clade 2B (Su). To contextualize our results within the *Serratia* population structure, we conducted a phylogenetic comparison of all available NCBI genomes (Fig. S3). We observed a highly similar distribution for available *Serratia* genomes, confirming that our strains represent the known diversity of human-associated *Serratia*.

#### Ecological and epidemiological relatedness

**Fig 3:**
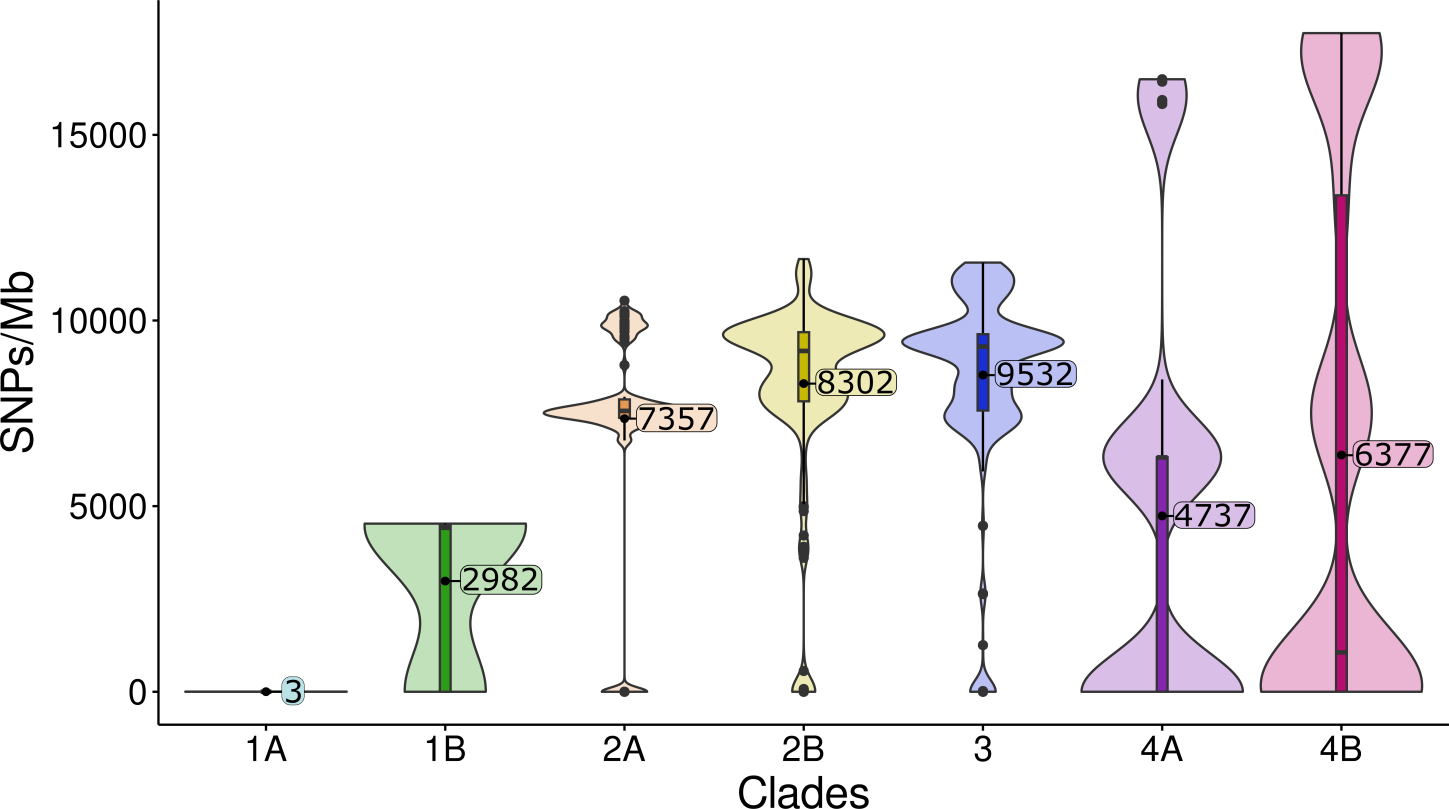
Violin plot showing the single nucleotide polymorphism (SNPs/Megabase [Mb]) distribution among the major clusters of the core *Serratia* genome. SNPs ranged from 35,533 to 45,719 SNPs/Mb between genomes of clades 1 and 2 (42,009 SNPs/Mb median distance). The significance values (Wilcoxon test + Bonferroni *P*-adjust) referring to comparisons of distributions of SNPs/Mb across subclades ranged from *P* < 0.001 (1A-2A, 1A-2B, 1A-3, 1A-4A, 1A-4B, 2A-2B, 2A-3, 2A-4A, 3-4A, 3-4B, and 4A-4B) to *P* < 0.05 (1B-2A, 1B-2B, 1B-3, 1B-4B, and 2A-4B). Comparisons 1A-1B, 1B-4A, and 2B-3 were not significant (*P* > 0.05).

The single nucleotide polymorphisms (SNPs/Mb) observed in the core genomes of clades and subclades are shown in Fig. 3. A diversity of *Serratia* populations (same and different subclades) coexist in our hospital, although clade 3 (Sm/Snv) and clade 4 (Snv) predominate in our study. We also identified the sequence types (STs) assigned to these genomes after querying the PubMLST database (27). Clade 3 (0–11,560 SNPs/Mb, median of 9,297 SNPs/Mb; 27 STs) is comprised of highly related isolates from 4/14 ICU sinks and patients (ST525, 0–7 SNPs/Mb, 2015–2020) collected 5-year apart along distinct genomes of 36 clinical strains (2003–2020). Some of these clinical isolates corresponded to STs long recovered in different wards (ST366, 2004–2011; ST394, 2004–2012; ST408, 2011–2015), each one represented by three isolates differing in 5–13 SNPs/Mb. Within clade 3, several STs are widely represented in the PubMLST database, and all strains showed the AmpC inducible phenotype (AMC^R^ + FOX^R^ + 3 GC^S^). Subclade 4A (0–16,498 SNPs/Mb, median of 4,736 SNPs/Mb, 5 STs) mostly encompasses highly related genomes from isolates with AmpC basal-like expression. They were assigned to ST92 (*n* = 23, 0–3 SNPs/Mb; isolates from 11/14 sinks carrying an AmpC and two clinical isolates collected in 2015 and 2016, one carrying a plasmid harboring *bla*_VIM-1_) and ST470 (*n* = 11, 0–21 SNPs/Mb; isolates from 7/14 sinks). Three STs correspond to unrelated genomes from clinical isolates (2004–2019). Subclade 4B (0–17,740 SNPs/Mb, median of 6376 SNPs/Mb, 10 STs) comprises both unrelated (15 BSI isolates with the inducible AmpC phenotype, distinct STs, 2004–16) and clonally related environmental and clinical (outbreak) VIM-producing isolates with an extended-depressed-like phenotype (ST424, 0–5 SNPS/Mb, 2017–2020). This epidemic clone was collected from 7/14 ICU sinks for at least 18 months during different outbreaks and persists to date (data not shown).

Subclade 2B-Su (10–11,656 SNPs/MB, median of 9,180, 22 STs) includes five environmental isolates (ST485, ST465, and ST496) from 4/14 ICU sinks with both the AmpC basal-like and inducible phenotypes and 21 clinical isolates (18 STs). Subclade 2A-Sb only accounts for clinical isolates (6,729–10,251 SNPs/Mb, median of 7,565, 13 STs), three of them clonally related (ST31, 0–1 SNP/Mb, 2004) (Fig. S2B; Table S2). Subclade 1A-Sn (ST431, 5 SNPs/Mb, 2004–2007) and subclade 1B-Sm (3 STs) were less represented in our sample, comprising only clinical isolates. Most of the STs described in this study were newly assigned by the curators of PubMLST.

### The accessory genome

In order to assess the presence of adaptive traits in different groups, we analyzed the accessory genomes of the 165 *Serratia* sequences using the Pangenome Analysis Toolkit (PATO) (28) (see Materials and Methods for details).

**Fig 4:**
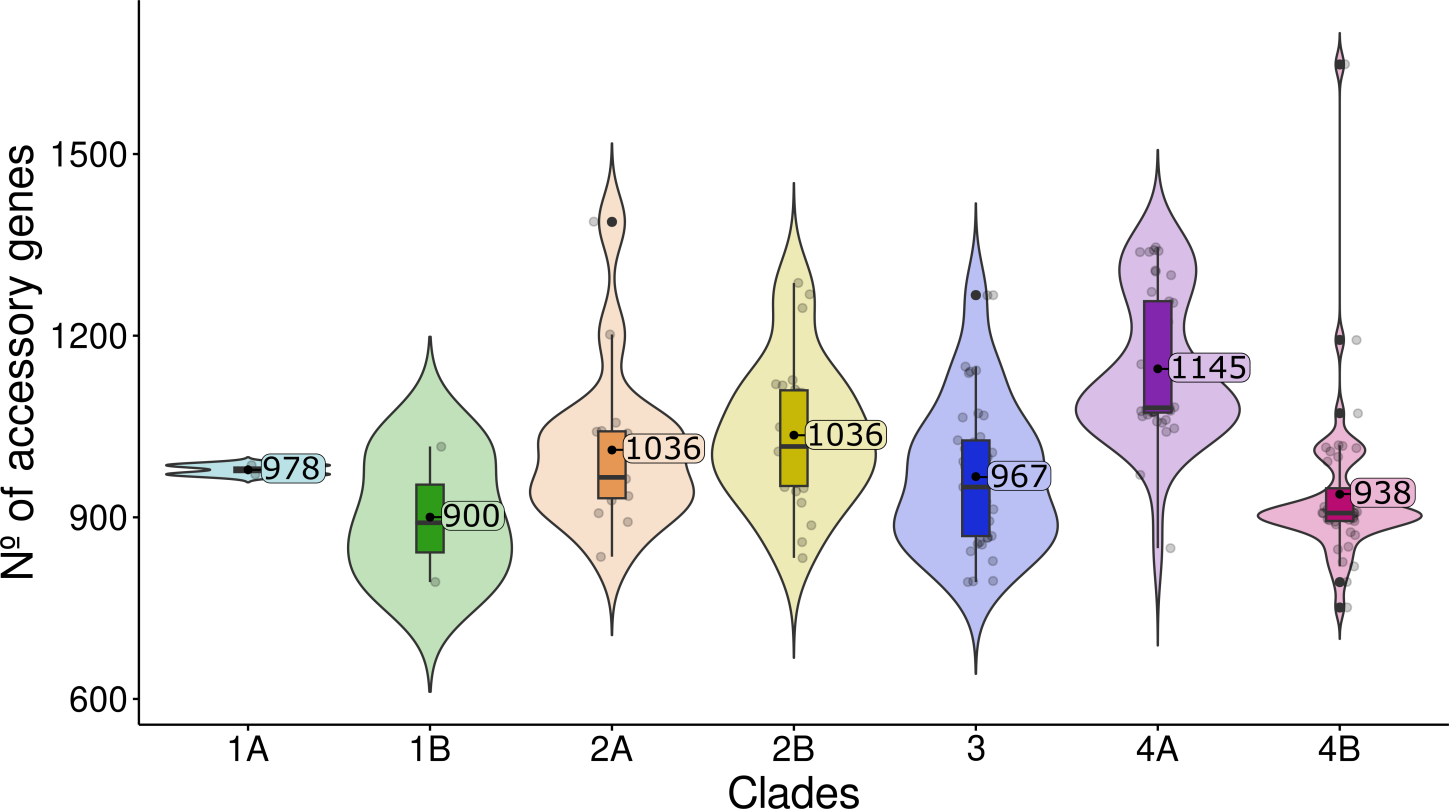
The accessory genome of the *Serratia marcescens* complex. The number of total shared genes among the clusters (sharedness) was calculated for each genome of each cluster and its mean value, employing the Pangenome Analysis Toolkit accent package (28).

We identified 9,307 accessory genes shared in less than 80% of the sequences. Figure 4 shows the average number of accessory genes per strain within each subclade. Several genes were significantly enriched within particular subclades (*P* < 0.001) (Table S3), including DNA-binding proteins, ATP-binding cassette (ABC) transporters, transcriptional regulators, oxidoreductases, and enzymes involved in lipid metabolism and fimbrial biogenesis. Of particular interest was the enrichment of RedY in subclade 1B (Sm), which is a homolog to HapK and is involved in the production of prodigiosin, a characteristic feature of *S. marcescens* to date (29). Additionally, isolates of clade 3 (Sm/Snv-like) and subclade 4B (Snv) were enriched in the toxin/antitoxin (TA) systems HigB/HigA and HicA/HicB, respectively, while subclade 4A (Snv) only contained antitoxin HigA. These TA systems are enriched in free-living bacteria such as *Pseudomonas* and are associated with adaptation to stress responses, biofilm formation, and persistence in host and non-host environments (30).

A Mantel test examined the correlation between the variables “ward” (the hospital ward in which either clinical or environmental isolates were recovered) and “origin” (the isolation source from a clinical or environmental sample) in relation to the phylogeny structure of the core and accessory trees. In the core-based tree, the variable “ward” correlated better with the phylogeny (*r*^2^ = 0.2119) than the variable “origin” (*r*^2^ = 0.1463). In the accessory-based tree, the variable “origin” (*r*^2^ = 0.3752) was better correlated with sample distribution than the variable “ward” (*r*^2^ = 0.2114). All the comparisons made were found to be statistically significant (*P* < 0.001).

### The resistome

A thorough analysis was conducted to determine the intrinsic and acquired resistome of SMC populations, which include genes that confer resistance to antibiotics, heavy metals and biocides.

#### The intrinsic resistome

All the strains contained chromosomally located genes linked to resistance against various antibiotic families, such as β-lactams (*bla*_AmpC_), aminoglycosides (*aac(6′)-Ic*), tetracyclines (*tetA*) (17, 31, 32), and polymyxins (*pmrA*). Furthermore, efflux pumps belonging to the resistance-nodulation-division (SdeAB, SdeXY, and OqxAB), ABC (SmdAB), and small multidrug resistance (SsmE) families, which are associated with resistance to a variety of antibiotics and/or biocides, were detected in all isolates (33–35) (Fig. S4).

Genotypic and phenotypic variability for specific housekeeping *Serratia* genes was observed among isolates of different SMC clades. Those encoding the AmpC cephalosporinase (and all of their regulatory proteins AmpR, AmpD, AmpE, and AmpG) were present in all the genomes; however, mutations in AmpC, AmpR, and AmpD were specific to the different clades as observed for other species of *Enterobacterales* (19) (Table S4; Fig. S6). In addition to the highly conserved AmpC motifs (64SXSK, 150YXN, and 315KTG) and other conserved residues linked to the reference AmpC β-lactamase of *Serratia* SRT/SST, we observed amino acid variations at positions N86K and R91H located in the middle of an alpha-helix (H) and a bend or turn (36, 37). Some mutations (GC > AT) in the intergenic region of *ampC*/*ampR* genes were observed (38) (Fig. S5). The *ant(3*″)-Ia, previously considered part of the intrinsic resistome, was only detected in clade 4 (Snv). All isolates but those of clade 3 (Sm/Snv-like) contained the allele *tet*41.

#### The acquired resistome

**Fig 5:**
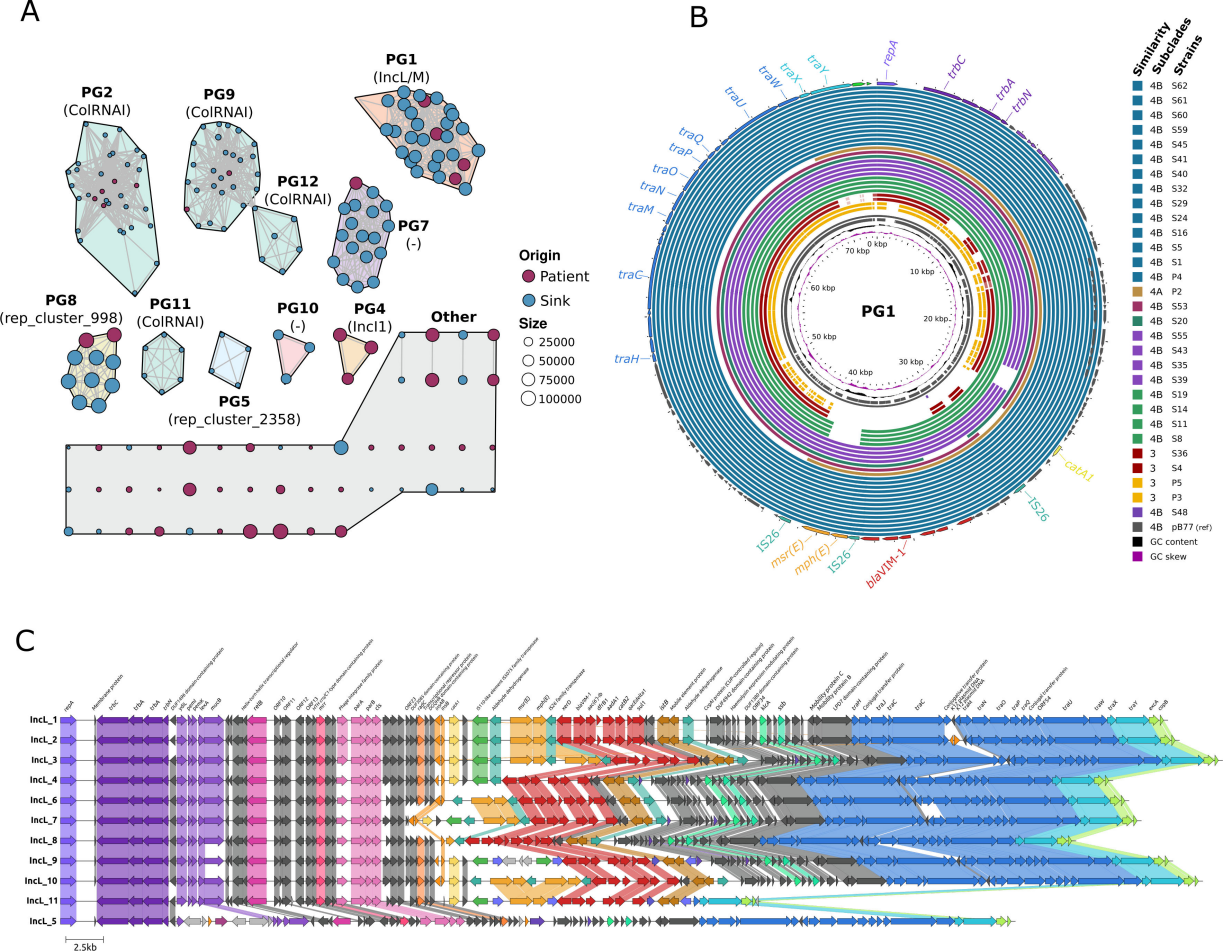
The plasmidome of the *Serratia marcescens* complex. Panel A: plasmid network built with the k-nearest neighbors’ algorithm (K-NNN) using the Pangenome Analysis Toolkit K-NNN function. Each node represents one plasmid and is colored according to the source (patient vs sink), its molecular weight being proportional to the node size (see keys). A plasmid group (PG) was defined using the Louvain algorithm over the network structure, each plasmid being connected with its best hits (Jaccard similarity >0.5, coverage/size difference <50%). PGs were arbitrarily designated with a number; their replicon content as defined by the MOB-typer tool (40), is shown below the PG label in brackets. Panel B: BLAST Atlas of *S. nevei* IncL plasmids in PG1 compared with IncL epidemic plasmids isolated in our institution. BLAST was applied to the coding sequences (CDS) within the reference plasmid from this group, obtained through annotation via Bakta of one of the outbreak plasmid strains (pB77, P6 strain) against sequence regions in the query plasmids. The circularized map of the plasmids was rendered with Gview (https://server.gview.ca/). The inner slots represent the guanine-cytosine (GC) skew, GC content, and CDS regions of the reference. The colored slots represent plasmid data from PG1 strains, mostly from subclades 3, 4A, and 4B. Panel C: pairwise alignment of all genes using clinker (41) with GenBank files (.gbff) among 11 variants of the IncL plasmid sequences obtained through MinION technologies and annotated with Bakta. A 100% identity is used for cluster similarity.

The isolates clustered in subclade 4B (ST424) carried a class 1 integron comprising genes that encode resistance to carbapenems (*bla*_VIM-1_), aminoglycosides (*aac(6′)Ib*), chloramphenicol (*catB2*), trimethoprim (*dfrB1*), sulfonamides (*sulI*), and quaternary ammonium salts (*qacEΔa1*) (Fig. 2). This gene array (*int*I1*-bla*_VIM-1_*-aac(6′)Ib-dfrB1-aadA1-catB2-qacEΔ1/sul1*) was observed in our hospital on a variety of plasmids and species of *Enterobacterales* for years (22, 25). The IS*26-msr(E)-mph(E*)-IS*26* transposon conferring resistance to macrolides and triamilides but not lincosamides [and widely spread in the plasmids of *Enterobacterales* and water systems (39)] was detected upstream from the class 1 integron (Fig. 5C). Only one other carbapenemase gene, *bla*_OXA-48_, was detected but was confined to clinical outbreak isolates. Of interest was the detection of *pcoABCDRS*, *silABCEFPRS*, and several genes of the *arsABCDR* and *mer* operons in the genomes of the clade 3 (Sm/Snv) and subclade 4B (Snv) (Fig. S4).

### The plasmidome

Tables S5 and S6 show the features of the 190 plasmids found among the 165 fully sequenced genomes. Of these 190 plasmids, 142 were clustered in 10 plasmid groups (PGs), and 48 were singletons (Fig. 5A). The predominant PGs in sinks corresponded to known plasmid families L/M (PG1) and Col (PG2, PG9, PG11, and PG12). IncF-like (PG7 and PG8) or plasmid not typed by the MOB-suite or PlasmidFinder (i.e., PG10, “others”) were also common in our collection.

Of special interest are PG1 and PG8, which appear to be transferable between sink SMC populations. The PG1 comprises plasmids of high epidemiological value belonging to the L and M families (42, 43). PG1 is overrepresented by a predominant and highly conserved IncL plasmid carrying the class 1 integron mentioned above (Fig. 5B), which was highly similar to pB77-CPsm recovered from *Serratia* outbreak isolates in our hospital since at least 2017 (22). Besides clinical and sink isolates of clade 3 (Sm/Snv) and clade 4 (Snv), this plasmid was also detected in isolates of *Klebsiella pneumoniae*, *Klebsiella variicola*, *Citrobacter cronae*, and *Enterobacter roggenkampii* collected for at least 5 years (2017–2022) from patients (25) and sinks (44). These ~70 kb pB77-CPsm variants differ in 0–8 SNPs and/or small indels, rearrangements, or duplications. They were also highly similar to the pOXA-48 circulating in our hospital (99.9% identity and 91% coverage) (23) encoding *bla*_OXA-48_ instead of the *bla*_VIM-1_ integron (Fig. 5C). It is of note that an IncL plasmid variant (~46 kb) from a clinical isolate, lacking a 27,125-bp region encoding genes of the conjugation module and categorized as a singleton (“other PGs”), was highly similar to PG1. PG1 also grouped an approximately 81-kb IncM1 plasmid (53.0% GC content) carrying tetracycline resistance genes (*tetA* and *tetR*) in sink isolates similar to a plasmid previously described in *E. coli* (45).

The PG8 comprises approximately 107-kb IncF-like plasmids highly alike in sink and patient isolates of subclade 2B (Su), clade 3 (Sm/Snv-like), and subclade 4B (Snv). These plasmids are almost identical to p87710 (96.8% identity, 60.0% coverage), an approximately 87-kb plasmid found in *S. marcescens* from an oligotrophic pond (GenBank: CP063230.1) but also contained a cluster of genes associated with fimbrial biogenesis (Fig. S7). PG7 encompasses plasmids of variable sizes found in our sink and patient isolates that are highly similar to pE28_003, a 67-kb plasmid isolated from a clinical strain of *S. marcescens* isolated in Australia (GenBank: CP042515) (Fig. S8). Various Col PGs (PG2, PG3, PG11, and PG12b) were also represented by sink and patient isolates of clades 3 and 4. Plasmids containing replicons of the plasmid incompatibility I1 group were categorized as PG4 and singletons, but they were only detected in clinical isolates.

## DISCUSSION

This work sheds light on the ecology and epidemiology of the “*S. marcescens* complex” and reveals the wide genotypic and phenotypic diversity of human-associated *Serratia* within the sinks of a single ICU ward. Such heterogeneity defines the population diversity of *S. marcescens* at both local and global levels. The findings of this study may have significant implications for diagnosing, managing, and treating infections caused by SMC species as well as for mitigating antimicrobial resistance in the healthcare system.

Firstly, a metapopulation of *S. marcescens* is part of the hospital’s environmental microbiome. This study confirms that well-known “*Serratia* species” such as *S. marcescens*, *S. bockelmannii*, *S. ureilytica*, *S. nematodiphila*, and the recently described *S. nevei* (16) belong to the SMC (14) and documents their coexistence in the hospital setting for the first time. From an eco-evolutionary perspective, the specific adaptive traits in the core and accessory genomes found for each SMC clade/subclade (“species”) reflect the diversification and adaptation of “*S. marcescens”* to disparate conditions, explaining the survival and coexistence of discrete populations in a given heterogeneous environment such as the hospital. We found a predominance of clade 3 (Sm/Snv) and clade 4 (Snv) in our local sample and in publicly available genomes lacking prodigiosin, as noted previously for some *Serratia* clades (14) or exhibiting unusual β-lactam susceptibility patterns. This raises questions about the assumed classical features of *S. marcescens* in contemporary human-associated SMC populations and the need to revise the taxonomic classification of SMC, particularly relevant for epidemiological purposes. Note that rMLST (27) was inadequate for accurately categorizing *Serratia* at the species level. Furthermore, the standardized microbial taxonomy based on genome phylogeny for bacterial populations with very high ANI values (>95%), such as SMC clades 3 and 4, depends on the reference genome selected for the analysis. We found *S. nevei* to be one of the most identified species based on ANI in human-associated settings (clades 3 and 4). Previously, *S. nevei* had been rarely identified in Germany and the US (16, 45) and is still rarely present in available databases, probably due to the lack of knowledge of the population structure of SMC and the misidentification of *S. marcescens*. This highlights the importance of further specifying the clade for epidemiological purposes because of relevant differences of clinical concern.

Secondly, the occurrence of *Serratia* in hospitals follows a “source-sink” dynamics (4). Hospital ICU sinks are increasingly identified as reservoirs of multidrug-resistant *Pseudomonas* and *Enterobacterales*, with most available studies focused on identifying the origin of outbreaks or the impact of interventions to control bacterial transmission (10, 11, 46–48). However, it is difficult to fully understand the dynamics (emergence, transmission, and maintenance) of opportunistic bacterial pathogens like *Serratia* (1) and antibiotic threats such as carbapenemase genes (49) in the hospital setting in the absence of long-term analysis of microbial populations. Our study uncovered a high diversity of clade 3 (Sm/Sn-like) populations from patients, some detected in both patients and sink, such as ST525. Furthermore, we found the coexistence of different long-term persistent clade 4 (Sn) clones in sinks able to cause HAIs. For example, an epidemic new ST424 (subclade 4B) producing VIM-1 as well as highly susceptible ST470 and ST92 clones (subclade 4A), the last one involved in a polyclonal outbreak of VIM-1 producers in 2017 after acquiring the IncL/pB77-CPsm plasmid (22). Although the directionality of transmission is always difficult to demonstrate, the persistence of clades 4A and 4B strains in sinks for more than 5 years (high recovery during and soon after outbreaks and low recovery when the ICU space was cleared and transformed for other non-healthcare activities) highlights the ability of *Serratia* to survive in non-host environments, an aspect only scarcely analyzed for certain *Enterobacterales* (50). There, they can acquire and serve as reservoirs for highly conserved and persistent multidrug-resistant plasmids. From the eco-evolutionary perspective, the limited clonal diversity of persistent *Serratia* clones in ICU sinks suggests that microbial interactions in these “abiotic patches” constitute “evolutionary dead-ends” of *Serratia* from patients and/or staff. Nonetheless, this situation could change according to the variability of the hospital environmental conditions that define such an ecological (Hutchinsonian) niche.

Thirdly, the AmpC β-lactamases of SMC clades show a wide sequence and phenotypic variability. Basal-like expression of AmpC β-lactamase leads to natural full susceptibility to aminopenicillins and cephalosporins, which is rare in species of the ESCPM group and has not been documented in *S. marcescens* (18, 19, 51). The reasons explaining the basal AmpC expression remain elusive, as we cannot associate this phenotype with any significant change in the sequences analyzed (AmpC, AmpD, and AmpR). Mutations in the *ampC/ampR* intergenic region may affect a large 5′UTR preceding the *ampC* coding sequence. Such mutations in the secondary structure might result in a rapidly degraded transcript, therefore, in lower rates of AmpC production (38). Nonetheless, a similar basal expression phenotype has just reported in *Enterobacter cloacae*, following inactivation of AmpG permease (52). The association of this basal-like phenotype with the predominant SMC clade 4 is of clinical importance due to the low probability of AmpC β-lactamase derepression upon therapy. This finding agrees with recent clinical studies that reported low rates of AmpC induction in hospitalized patients infected by *Serratia* (although without establishing any link with the microbial taxonomic background causing such infections) and reinforces the need to accurately identify them and test the antibiotic susceptibility prior to treatment implementation (20). The results would also suggest that AmpC and/or the proteins of its induction cascade would have a role in the adaptation to non-host habitats. Enzymes involved in peptidoglycan cycling, such as PcgL and DdpX, have been involved in survival outside humans (50). In *P. aeruginosa* and *Stenotrophomonas maltophilia*, AmpR regulates β-lactamases, proteases, quorum sensing, biofilm formation, and other virulence factors (53–55). The clade specificity of AmpC β-lactamase reflects an ancient diversification of the SMC. Notably, the variability of sequences within each clade is probably attributed to exposure to multiple and independent selection events in the hospital, but we cannot discard genetic drift (including recombination).

Fourthly, this work sheds light on the role of *Serratia* in the dynamics of clinically relevant antibiotic-resistant genes and plasmids. The plasmidome of the SMC is still poorly explored, with a few reports only describing the diversity of plasmid replicons or the carriage of multidrug-resistant plasmid by a small number of *S. marcescens* clinical isolates during outbreaks (8, 33, 56). The long-term persistence of highly conserved IncL epidemic plasmids carrying *bla*_VIM-1_ or *bla*_OXA-48_ by sink isolates of *Serratia* and other species of the ESPCM group reveals a microbial community that provides population-wide access to broad-host plasmids. This finding contributes to explaining the endemicity of *bla*_VIM-1_ and *bla*_OXA-48_ and plasmid evolution in hospitals far beyond the detailed description of the “units of selection” (clones, plasmid, integrons, and transposons) provided by cross-sectional multicentric studies or local reports of polyclonal outbreaks (23, 57). In addition to the IncL plasmids, the distribution of different families of plasmids in sink and clinical isolates (e.g., Col, IncI1, and IncF-like plasmids) mirrors the process by which this species easily acquired, maintained, and enabled plasmid evolution efficiently in various “hospital patches,” both “source” (permanent) and “sink” (transient) habitats.

In conclusion, the abiotic reservoirs (sinks) of the ICU environment account for diverse and coexisting populations of the SMC able to survive for years. Certain SMC clades constitute a unique reservoir of plasmid-carrying genes encoding carbapenemases that can facilitate the endemicity and unpredictable emergence of nosocomial outbreaks involving this species and/or plasmids. From an eco-evolutionary perspective, the findings reflect a “source-sink” dynamic model for SMC lineages and plasmids. The model, used in ecology to understand variations of population growth in heterogeneous habitats such as the hospital ecosystem, has been used to explain antimicrobial resistance by only a few reductionists *in vitro* studies using single plasmids or single clonal backgrounds (4, 58). Here, we demonstrate the relevance of hospital patches (network structure) in the epidemic propagation of clones and plasmids, which is necessary to establish connectivity-dependent infection schemes and to explore the threshold effects where infections would otherwise be unexpected (5).

## MATERIALS AND METHODS

### Study design and sample collection

The Ramón y Cajal University Hospital is a tertiary-level public health center with 1,155 beds, which provides attention to 600,000 inhabitants in the northern area of Madrid (Spain). A 34-month prospective study in its largest ICU ward (14 rooms and 2 monitor areas) was conducted to identify possible environmental reservoirs of opportunistic pathogens. We included 2,417 samples recovered from sinks (surface, drain, and p-trap, *n* = 1,126) and room surfaces (bed rail, ventilator touchscreen, and pot, *n* = 1,291), which were collected weekly (with some discontinuations due to the SARS-CoV-2 pandemic). The study comprised three periods according to the ward occupancy and the functional uses of the hospital ward. Periods A (March 2019–February 2020) and B (July 2020–February 2021) covered the ICU occupancy before and during the SARS-CoV-2 epidemic, respectively, and period C (March 2021–December 2021) covered the clearance of patients to adapt the ICU ward to other hospital uses and transferred to another floor. The ICU was moved to another hospital floor in 2021, and another sampling analysis was started (data not included in the study).

Sink surfaces were sampled with polyurethane sponges placed in a sterile bag impregnated with 10-mL HiCap Neutralizing Broth (EZ-10HC-PUR, EZ Reach Sponges, World Bioproducts, Bioing sro, Czech Republic). Sink drainage and p-trap samples were collected with standard sterile swabs and aspiration probes, respectively. The samples were plated onto BD CHROMagar Orientation Medium, BD CHROMagar ESBL-Biplate, and mSuperCARBA (Becton Dickinson, Franklin Lakes, USA). The plates were then incubated for 48 h at 37°C. Colonies of different morphotypes (size, color, and shape) were subcultured onto BD CHROMagar Orientation Medium, incubated for 24 h at 37°C and further identified with MALDI-TOF MS (MALDI Biotyper, Bruker, Billerica, MA, USA). Stocks of sub-cultured bacterial isolates identified as *Serratia* species by MALDI-TOF-MS (reliability score >2) were stored at −80°C in 1,500-µL Luria-Bertani broth with 15% glycerol for further analysis.

In addition to the environmental isolates prospectively recovered in this study, we incorporated a sample of 99 clinical isolates for comparative analysis (59). They included 93 blood isolates causing individual episodes of BSI between 2003 and 2016, five clinical isolates (two broncho-aspirates, two wound exudates, and one bone sample) involved in hospital outbreaks of VIM-1 and OXA-48 producers between 2016 and 2018 (18), and one fecal isolate recovered during the prospective longitudinal study carried in 2019. Table S1 summarizes the epidemiological features of all isolates. For the plasmid analysis, we analyzed 25 isolates of different species of *Enterobacterales* carrying IncL-*bla*_VIM-1_ plasmids and involved in polyclonal outbreaks in our institution (21) or collected in contemporary studies related to the hospital environment (44). Clinical strains were isolated using Columbia Blood Agar with Sheep Blood Medium (Thermo Fisher Scientific, Waltham, MA, USA).

### Antibiotic susceptibility

We performed susceptibility testing by disk diffusion against 14 antibiotics for all 1,432 *Serratia* isolates and further interpretation of the phenotypic results following CLSI (60) and EUCAST ((https://www.eucast.org/mic_distributions_and_ecoffs/) guidelines. They included AMC (20/10 µg), ampicillin (10 µg), cefoxitin (30 µg, FOX), ceftazidime (10 µg), cefotaxime (5 µg), cefepime (30 µg), aztreonam (30 µg), temocillin (30 µg), meropenem (10 µg), ciprofloxacin (5 µg), chloramphenicol (30 µg), gentamicin (10 µg), tobramycin (10 µg), and sulfamethoxazole-trimethoprim (25 µg). Antibiotic susceptibility of clinical isolates was measured using MicroScan (Beckman Coulter, Nyon, Switzerland). The double-disk synergy test (AMC and cefotaxime-ceftazidime-cefepime-aztreonam) was performed to screen for ESBL production. Carbapenemase production was phenotypically suspected with the analysis of the β-lactam phenotype. AmpC β-lactamase induction was screened using cefoxitin and imipenem (medium and strong inducers, respectively) over aminopenicillins (ampicillin), 2GC (cefuroxime), and 3GC (cefotaxime, ceftazidime) as indicators (60). The presence of carbapenemase genes (*bla*_VIM-1_, *bla*_OXA-48_, *bla*_KPC_, *bla*_NDM_, and *bla*_GES_) was tested with a multiplex polymerase chain reaction assay (61).

### Clonal relationship

A clonal relationship between isolates was preliminarily established by PFGE, following the PulseNet website protocols (https://pulsenetinternational.org/protocols/pfge/). Comparison of XbaI-digested genomic patterns revealed distinct PFGE types (or pulsotypes) (>4 bands) from which we selected a set of isolates for further typing by whole genome sequencing according to spatiotemporal distribution, PTs, and antibiotic susceptibility.

### Genome sequencing

Most isolates were “*de novo*” sequenced by Illumina (66 sink isolates and 94 clinical isolates from the strain collection of the microbiology department), with the sequence of 23 isolates being further closed using long-read sequencing (Oxford Nanopore, Oxford, UK).

For short-read sequencing, we used the Chemagic DNA Bacterial External Lysis Kit (PerkinElmer, USA) following the manufacturer’s recommendations. DNA quality and concentration were measured in a NanoDrop 2000 Spectrophotometer (Thermo Scientific, Waltham, MA, USA) and Qubit 2.0 Fluorometer (Life Technologies, Waltham, Massachusetts, USA), and fragment length was assessed with the TapeStation 2200 (Agilent, Waldbronn, Germany). We performed fragmentation of 4 µg DNA in a 46-µL elution volume in Covaris G-tubes by centrifuging at 4,200–5,000 rpm (Eppendorf 5424 centrifuge) for 90 s to achieve fragment sizes of ~20  kb. We prepared DNA libraries employing the Nextera XT library preparation kit and the Nextera XT v2 index kit (Illumina, San Diego, CA, USA). The library was sequenced on a HiSeq4000 (Illumina) using the reagent kit v2 (Oxford Genomics Center, Oxford, UK) to generate 250-bp paired-end reads.

For long-read sequencing, DNA was extracted with the MagnaPure 96 System (Roche, Basilea, Switzerland) and quantified with a Qubit 2.0 Fluorometer. For the library preparation, we used a minimum concentration of 20 ng/µL and the Rapid Barcoding kit 96 (Oxford Nanopore Technologies, Oxford, UK), following the manufacturer’s instructions. The libraries were loaded onto flow cell versions FLO-MIN106 R9.4 MinION (Oxford Nanopore Technologies, Oxford, UK) and sequenced for 72 h. Base calling was performed in real time with Guppy integrated in MinKNOW (Oxford Nanopore). Adapter sequences were removed with qcat (Oxford Nanopore), and fastq reads were filtered and quality measured with NanoFilt (>10,000 bases) and Filtlong v0.2.039 (500 Mbp best reads) before the assembly. Hybrid assemblies were created with Unicycler v0.4.7 (62) and checked by visualization with Bandage v0.8.1.

### Genomic analysis

The 165 sequenced genomes were first annotated with Bakta 1.7.0 (63). The core and accessory genomes were then defined using the PATO, which enabled us to analyze the population structure, annotate adaptive features, and create gene networks (28). More specifically, we used the PATO functions “core_plots” (to calculate the size of the pangenome, core, and accessory genome) and the “similarity_tree” (to generate pseudo-phylogenetic trees).

#### Taxonomic and epidemiological typing

We assigned the species to each isolate using two approaches, the function “classifier” of PATO (28) and GTDB-Tk (64), a software of the Genome Database Taxonomy (GTDB) (https://gtdb.ecogenomic.org/). “Classifier” assigns each query genome to the closest reference genome from the NCBI (https://www.ncbi.nlm.nih.gov/taxonomy) by calculating the ANI of each genome to the reference ones. It assigns a reference species if the ANI is over 95% of identity. GTDB-Tk assigns each genome to one of the clusters created by the GTDB (https://gtdb.ecogenomic.org/) and to the genome they have selected as reference for the species. The accession numbers for the reference genomes used at the NCBI browser were GCF_003516165.1 (*S. marcescens*)*,* GCF_017309605.1 (*S. ureilytica*), and GCF_016742975.1 (*S. nevei*). The accession numbers for the reference genomes used at the GTDB were GCF_017299535.1 (*S. marcescens*), GCF_013375155.1 (*S. ureilytica*), GCF_008364245.1 (*S. nevei*), GCF_000738675.1 (*S. nematodiphila*), and GCF_008011855.1 (*S. bockelmannii*). The phylogenetic tree from Fig. S3 that included more than 3,000 sequenced genomes of *Serratia* from the NCBI was created using the function “similarity_tree” and the values from the function “mash” from PATO. Clades were defined based on minimum within-node pairwise ANI scores using the unsupervised clustering MClust (65). We also analyzed the genome sequences by multilocus sequence typing and rMLST using the scheme for *Serratia* spp. available at https://pubmlst.org/organisms/serratia-spp (27).

#### Core genome

We defined core genes with the “core_genome()” function from PATO, which identifies and generates a hard core genome alignment comprising genes present in 100% of genomes without repetitions, using the output of “mmseqs().” This function defines paralogous genes of the core genome using a rapid pseudo-multiple sequence alignment method, leveraging “mmseqs2’s result2msa” for protein sequences and a blast-based approach for DNA sequences. We built a maximum likelihood phylogenetic tree (Fig. 2) through a pseudo-multisequence alignment with FastreeMP (65) midpoint root. We removed the paralogues with Panaroo (40) and the high-density polymorphisms with Gubbins (66), and we calculated the molecular clock and the time frame of the tree with TemEST (67) and BactDating (https://github.com/xavierdidelot/BactDating). The tree was displayed and annotated in the R package using ggtree version 2.2.4. We employed iTOL (41) to edit and generate metadata linked to the tree.

#### Accessory genome

We defined the accessory genome as the set of genes detected in less than 80% of the genomes and determined it employing PATO with default parameters (80% identity and 80% coverage) (28). The accessory genes enriched in each subclade were further analyzed using the PATO function “accnet_enrichment_analysis,” which performs a multi-hyperparametric test to find overrepresented genes in a cluster compared with the whole population (i.e., the genome data set). The accessory network was illustrated with Gephi for rearranging and uploading the layout in R to plot the network.

#### Plasmid analysis

Plasmids were reconstructed from FASTA files, typed using the MOB-recon and MOB-typer modules of the MOB-suite software (68), and annotated with Bakta (63). A distance matrix using gene-by-gene presence-absences using the “accnet” function of PATO with a default similarity parameter of 70% was created using Jaccard similarity. Then, we generated a k-nearest neighbor network (K-NNN) to allow reciprocal connections with the thresholds of 10 neighbors and 0.5 Jaccard distance. This implies that any plasmid is linked to its 10 most similar plasmids provided they share more than 0.5 Jaccard similarity. Plasmids were clustered from the K-NNN using mclust (65).

The plasmid network was built with Cytoscape, imported to R with the tidygraph R package, and set with the Louvain cluster algorithm (igraph R package). Information about plasmid features (predicted mobility, replicase, and relaxase IDs) was added to the network. Plasmid distribution over phylogenetic trees was built with the ggtree R software. All data manipulation and visualization were performed with the tidyverse R meta package.

The categorization of replicases and genes encoding resistance to antibiotics, biocides, and heavy metals was performed by submitting hybrid assemblies of plasmid sequences to Plasmidfinder (39), Resfinder (69), and BacMet databases (70). Gview (https://server.gview.ca) was employed for generating the circular map, and clinker (71), for the linear visualization and gene cluster comparison.

### Statistics

The Mantel test implemented in R was employed to compare the structure of the core and accessory trees by determining the correlation between the distance matrices. A nonpaired Student’s *t*-test (stats R package) was applied for the significance test of the number of accessory genes per genome. We used a statistical test to analyze the shared accessory genes within each clade/subclade, modeling the distribution of shared gene pairs between genomes in each group. This analysis was further corrected according to the similarity between genomes. To evaluate the association of the metadata variables (origin, collection place, and year) with the core and accessory tree, we employed the “envfit” function in the vegan R package. This function calculates the multiple regression of environmental variables with the ordination axes and estimates the significance with a permutation test. The *P*-values were adjusted based on the Bonferroni correction. The goodness-of-fit statistic is the squared correlation coefficient (*r*^2^), which measures the correlation of the variable with the ordination. We assessed the significance of the SNPs/Mb within each clade/subclade using a Wilcoxon test for each group comparison. The *P*-values were subsequently adjusted using the Bonferroni correction method.

## Data Availability

The sequences generated during this study were deposited in the European Bioinformatics Institute database under BioProject accession number PRJNA1023224 (accessible from 1 January 2024). Biosample accession numbers for each sequence can be found in Table S1. WGS data from outbreak clinical strains were previously submitted under the accession numbers JAAAMA000000000, JAAAMB000000000, JAAAMC000000000, JAAAMD000000000, and JAAAME000000000. Closed plasmids were fully sequenced and analyzed during this work under the same BioProject. Fig. S1 to S8 and Tables S1 to S5 provide information explained in the text.

## SUPPLEMENTAL MATERIAL

Supplemental Figures mbio.03054-23-s0001.pdf Figures S1 to S8.

Table S1 mbio.03054-23-s0002.xlsx Data set of the 165 genomes of *Serratia*.

Table S2 mbio.03054-23-s0003.xlsx SNPs per megabase of *Serratia* genomes in our study.

Table S3 mbio.03054-23-s0004.xlsx Accessory genome significantly enriched in each of the *Serratia* sublades.

Table S4 mbio.03054-23-s0005.xlsx Alignment of aminoacid sequences from AmpC, AmpR ,and AmpD consensus per sublade.

Table S5 mbio.03054-23-s0006.xlsx PG summary of the gene information and metadata.

Table S6 mbio.03054-23-s0007.xlsx Plasmidome data obtained from MOBTyper with the respective PG.

ASM does not own the copyrights to Supplemental Material that may be linked to, or accessed through, an article. The authors have granted ASM a non-exclusive, world-wide license to publish the Supplemental Material files. Please contact the corresponding author directly for reuse.
