# Supplementary material for: The ICU environment contributes to the endemicity of the “Serratia marcescens complex” in the hospital setting
Source: mBio. 2024 Apr 2;15(5):e03054-23. doi: 10.1128/mbio.03054-23 (PMC11077947; doi:10.1128/mbio.03054-23)
Supplement: Supplemental Figures — Figures S1 to S8. [file mbio.03054-23-s0001.pdf]

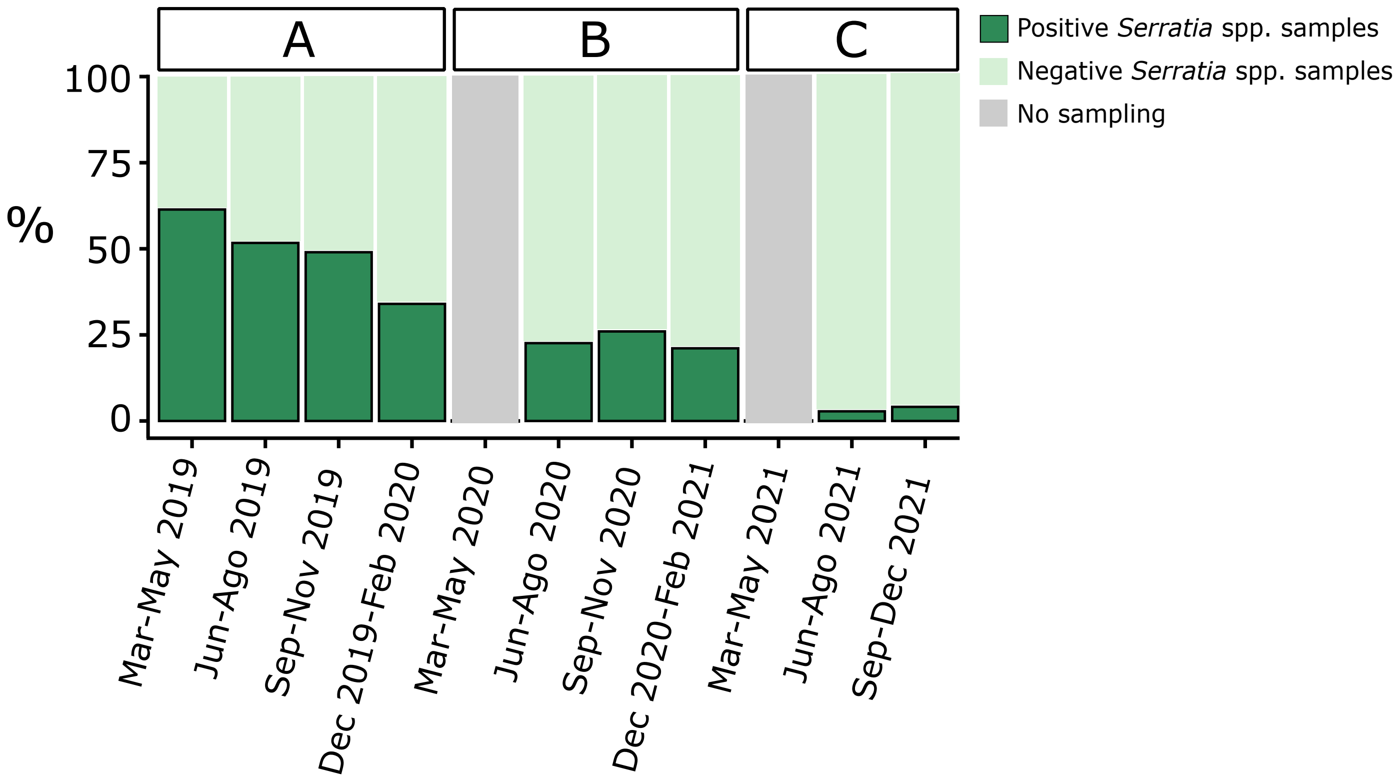

Fig. S1. Temporal variation of *Serratia* isolated from sinks. Positive samples for *Serratia* isolation from sinks across the three periods of study (A, B and C, see text) are represented.

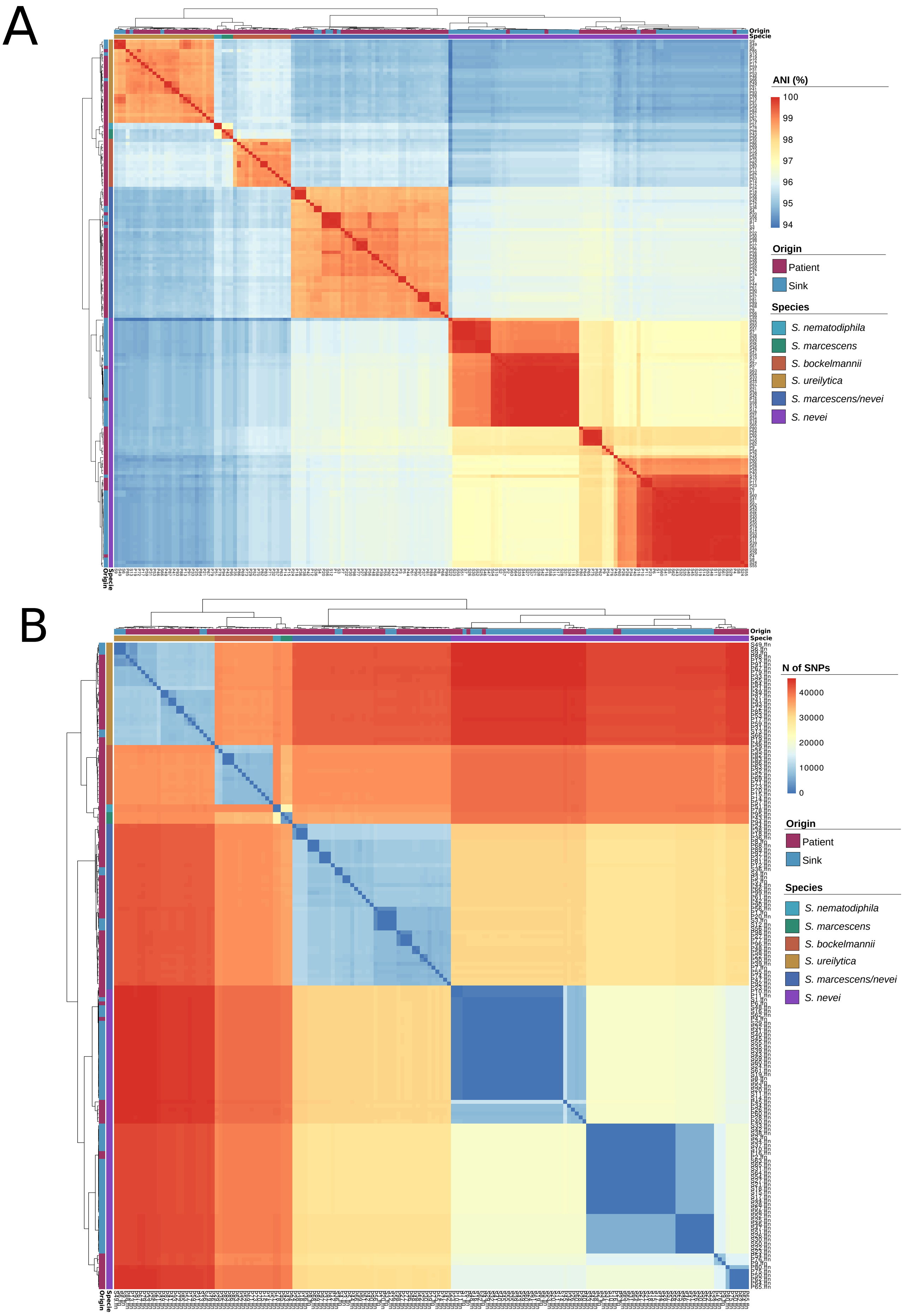

Fig. S2. Comparative analysis of *Serratia* isolates based on A) ANI and B) SNPs. Average nucleotide identity (ANI) and Single Nucleotide Polymorphisms (SNP) between *Serratia* isolates *S. nematodiphila*, *S. marcescens*, *S. bockelmannii*, *S. ureilytica*, *S. marcescens/nevei* and *S. nevei* from environmental and clinical origin represented by a heatmap in panels A and B respectively. The color bar represents the ANI or SNP value, whereas the lines bordering the figure represent the origin and species (from outside to inside).

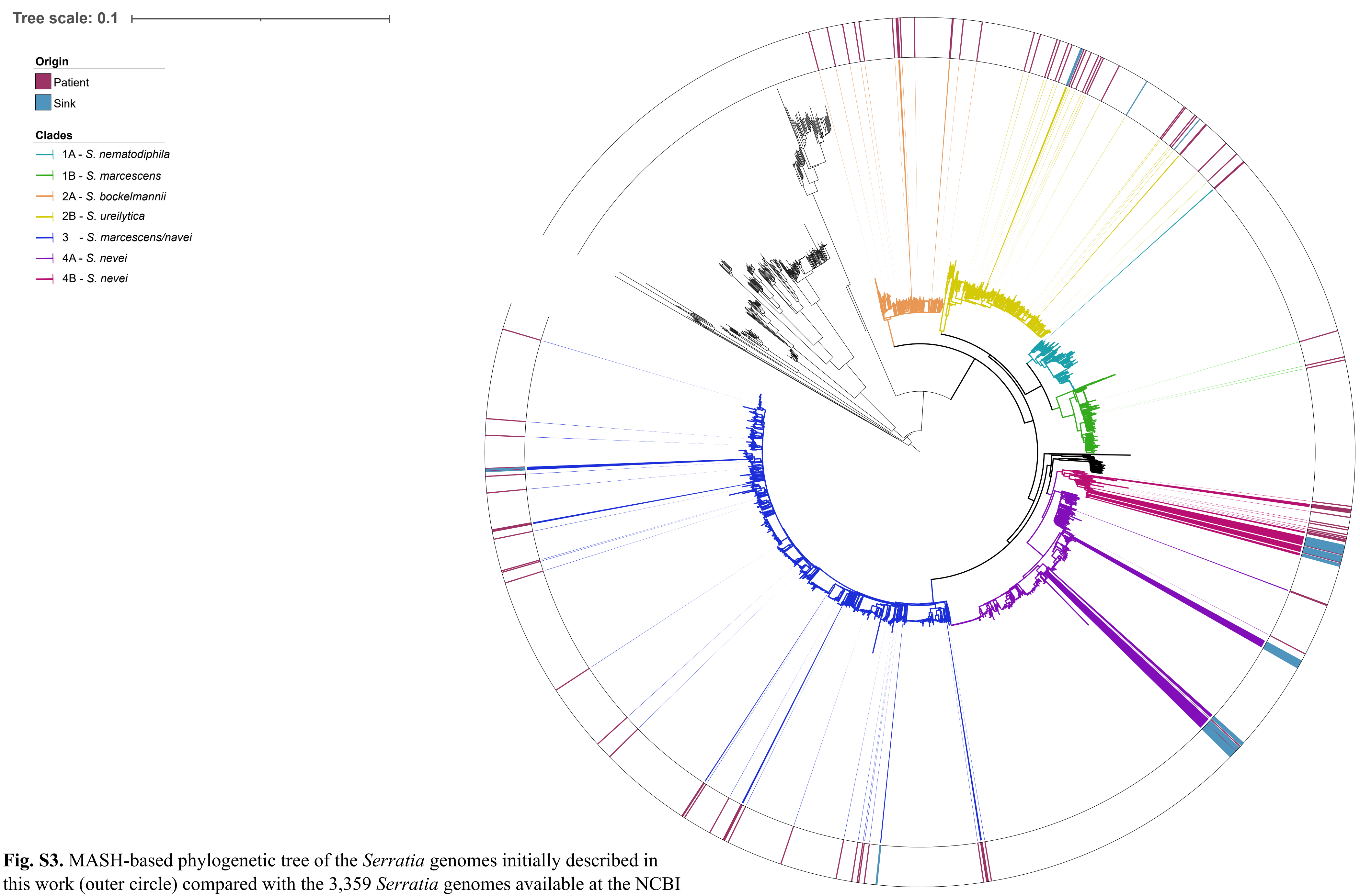

Fig. S3. MASH-based phylogenetic tree of the *Serratia* genomes initially described in this work (outer circle) compared with the 3,359 *Serratia* genomes available at the NCBI database on 12 January 2024. Clades and subclades from the core-based phylogenetic tree of *Serratia* are represented at branch colors (see key). The origin (patient/sink) is also indicated by different colors for our strains in the outer circle (see key).

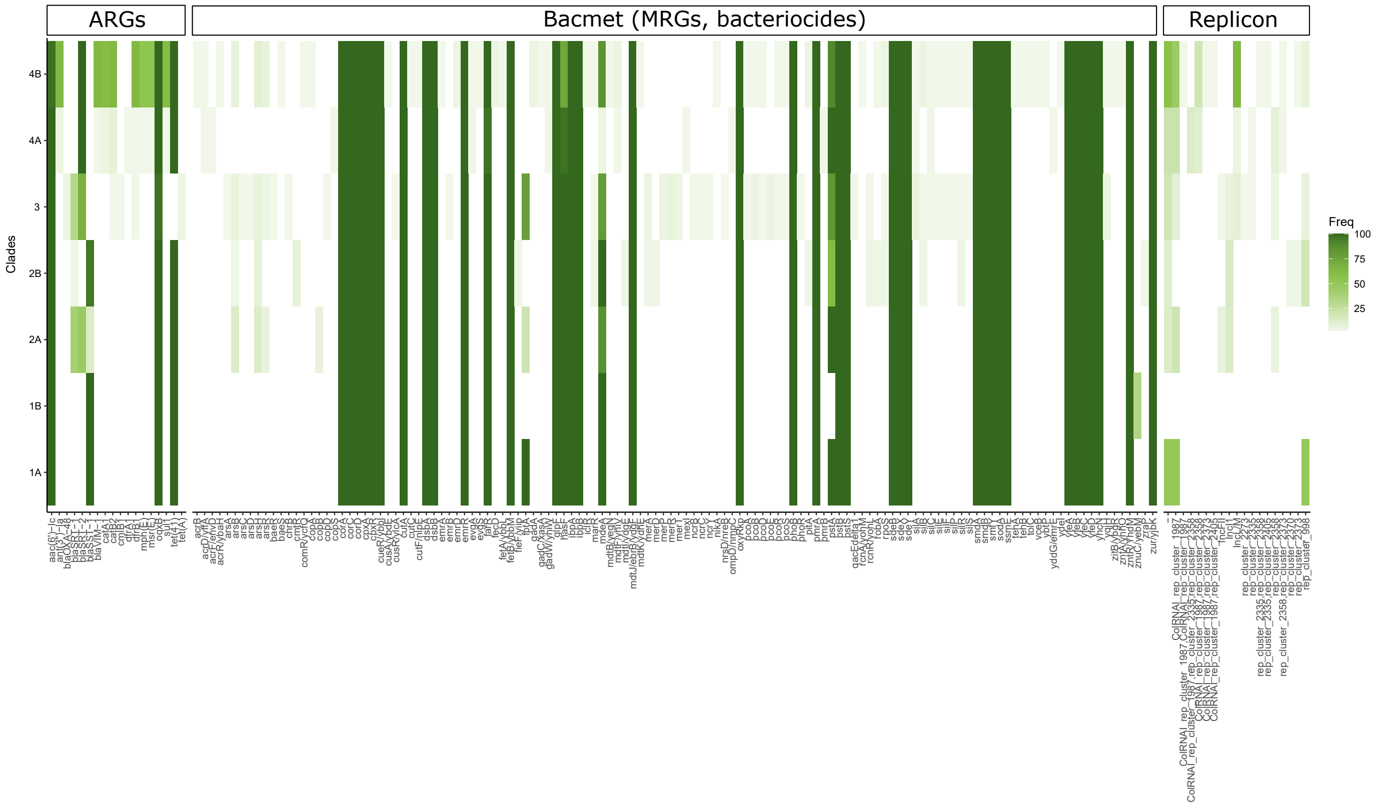

Fig. S4. **Resistome, metalome, and plasmidome of SMC isolates.** The abscissa shows the isolates grouped in the clades/subclades 1A (*S. nematodiphila*), 1B (*S. marcescens*), and 2A (*S. bockelmannii*), 2B (*S. ureilytica*), 3 (*S. marcescens/nevei*), 4A and 4B (*S. nevei*). The ordinate axis represents ARGs (antibiotic-resistant genes), MRGs (metal -resistance genes), and plasmid replicons. They were identified after interrogating the ResFinder (https://cge.food.dtu.dk/services/ResFinder/), BACMET (http://bacmet.biomedicine.gu.se/advanced_search.pl) databases, and MOBsuite databases.

```
Mahlen et al 2003                                    AGGGTATCGGCCGCGGCCAAAAGTTTTTATTGGCGTTATCGACGGCGCTTTCCCTG
Clade 3    CATGATAGAAGCTCTTAGGTTGCAGGGTATCAGCCGCGGCCAAAAGTTTTTATTGGCGTTATCGACGGCGCTTTCCCTGATTGATGATGTCATTCCTTTACGGCTGCGGCGACGCTCACAGCAGCCGCGCCAAATATCGGATTTTGCCGCCGGGTTGACAAGCGATAATTAATTTCAGCCAGCATAAAGAAAAACTTATGGCTATCGACAATG
Clade 4A   CATGATAGAAGCTCTTAGGTTGCAGGGCATCGGCCGCGGCCAAAAGTTTTTATTGGCGTTATATACGGCGCTTTCCCTGATTGATGATGTCATTCCTTTACGGCTGCCGTGACACTCTGAGCAGCCGCGCCAAATATCGGATTTTGCCGCCGGGTTGACAAGCGATAATTAATTTCAGCCAGCATAAAGAAAAACTTATGGCTATCGACAATG
Clade 4B   CATGATAGAAGCTCTTAGGTTGCAGGGCATCGGCCGCCGCCAAAAGTTTTTATTGGCGTTATATACGGCGCTTTCCCTGATTGATGATGTCATTCCTTTACGGCTGCCATGACACTCTAAGCAGCCGCGCCAAATATCGGATTTTGCCGCCGGGTTGACAAGCGATAATTAATTTCAGCCAGCATAAAGAAAAACTTATGGCTATCGACAATG
           ← AmpC   RBS   stem-loop   +1   -10 ampC   -35 ampR   -35 ampC   -10 ampR   A.........N11...........T   → AmpR
```

Fig. S5. Intergenic region from the *ampR/ampR*. Nucleotide sequence of the *ampC/ampR* intergenic region of complete genomes from isolates from Clades 3 and 4 in comparison with a reference (38).

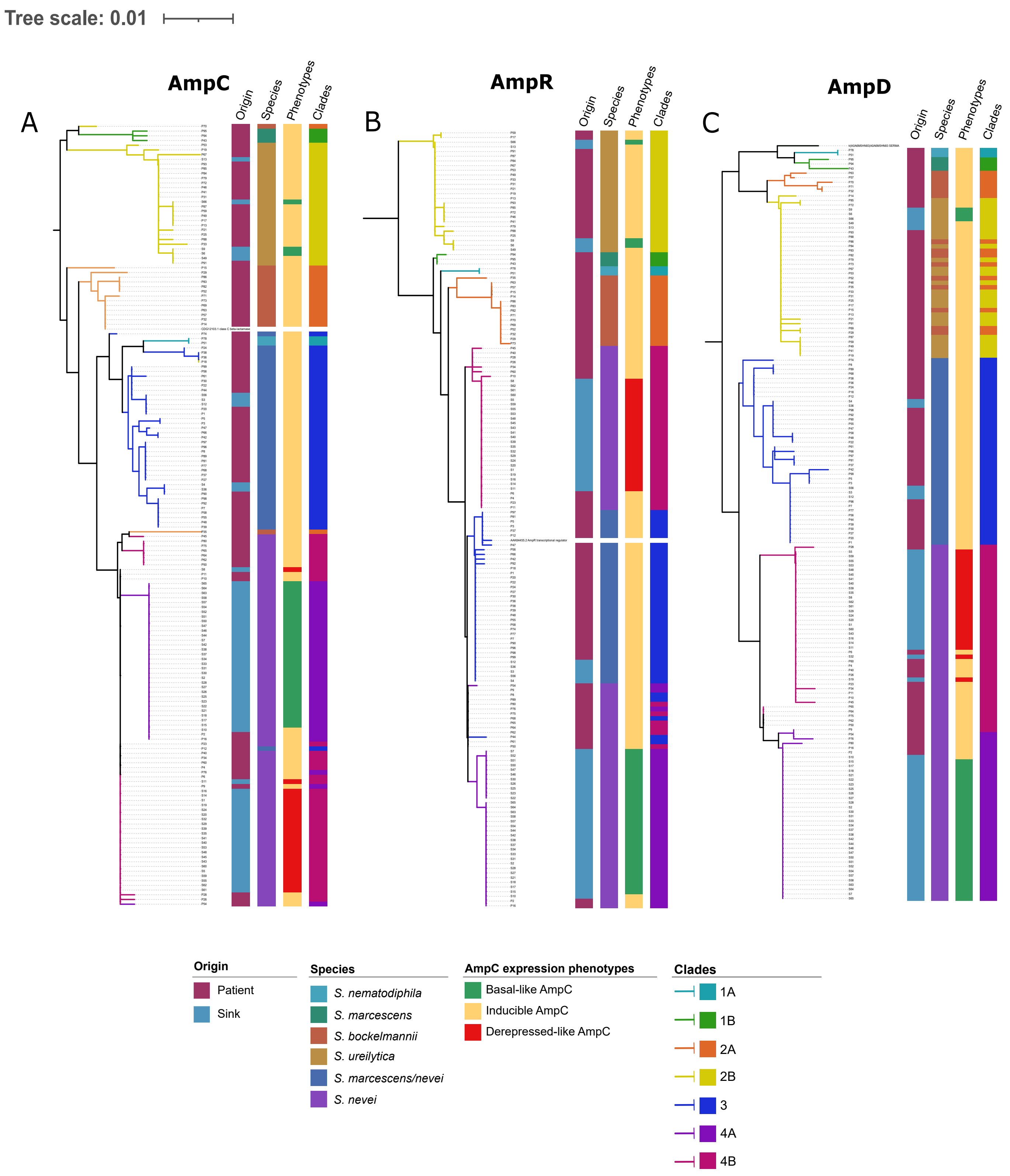

Fig. S6. Phylogenetic analysis of AmpC, AmpR and AmpD. Phylogenetic tree of a multiple sequence alignment of the AmpC, AmpR, and AmpD protein sequences of *Serratia* spp. (panels A, B, C, respectively) generated by ClustalW (https://www.ebi.ac.uk/Tools/msa/clustalo/). The epidemiological features of the isolates (β-lactam phenotype, sample origin, *Serratia* species and clades/subclades) inferred from the core genome phylogenetic tree are represented in bars (see keys).

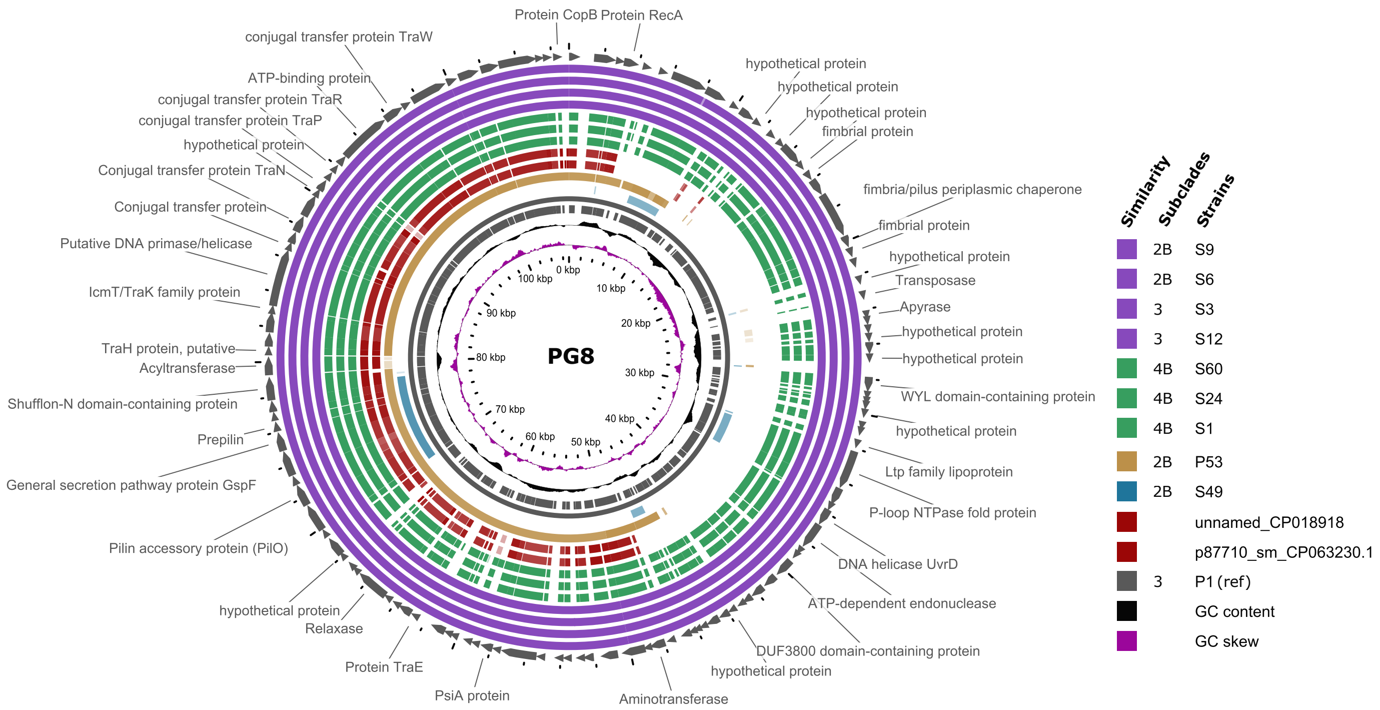

Fig. S7. Basic Local Alignment Search Tool (BLAST) Atlas of *Serratia* IncF-like plasmids in PG8. BLAST was applied to the coding sequences (CDS) within the reference plasmid from this group, obtained through annotation via Bakta (S60_plasmid2) against sequence regions in the query plasmids. The circularized plasmid map was rendered with Gview (https://server.gview.ca/). The inner slots represent the guanine-cytosine (GC) skew, GC content, and CDS regions of the reference.

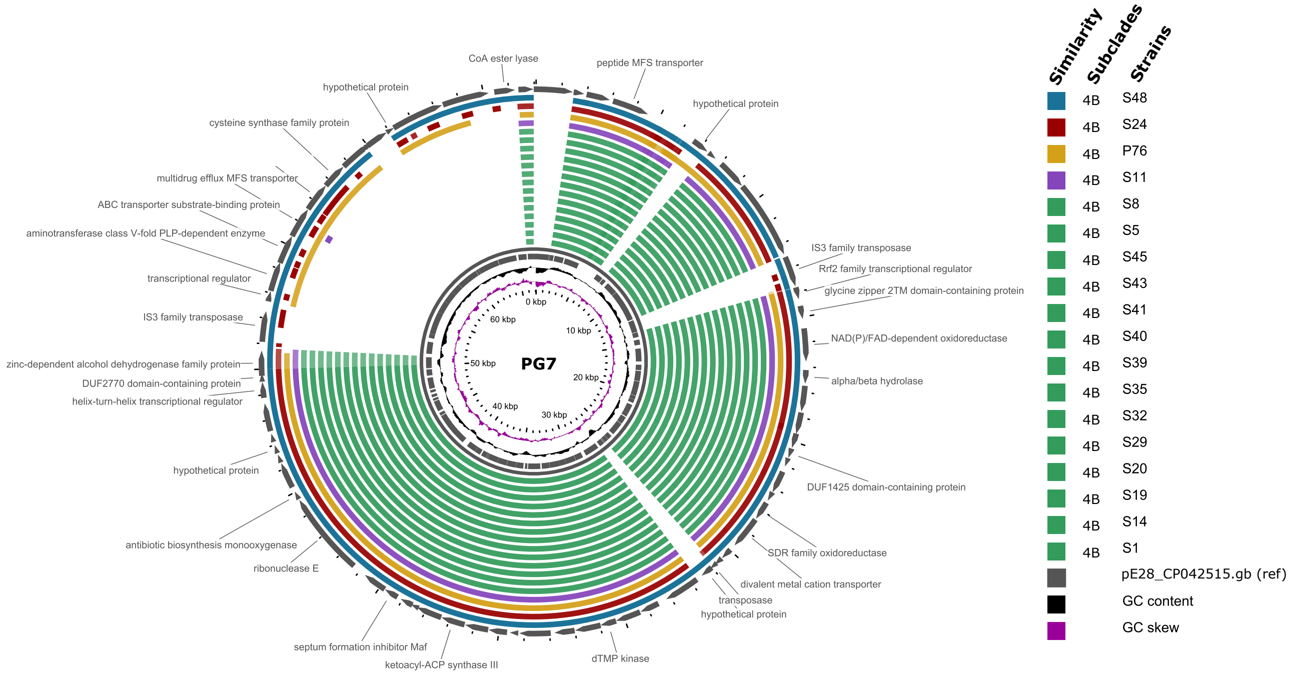

Fig. S8. Basic Local Alignment Search Tool (BLAST) Atlas of *S. nevei* plasmids in PG7. BLAST was performed to the coding sequences (CDS) within the reference plasmid from this group (pE28, GenBank: CP0425, most similar plasmid obtained through MOBTyper) against sequence regions in the query plasmids. The circularized map of the plasmids was rendered with Gview (https://server.gview.ca/). The inner slots represent the guanine-cytosine (GC) skew, GC content, and CDS regions of the reference.
